# RAB37 interacts directly with ATG5 and promotes autophagosome formation via regulating ATG5-12-16 complex assembly

**DOI:** 10.1038/s41418-017-0023-1

**Published:** 2017-12-11

**Authors:** Yue Sheng, Ying Song, Zhigang Li, Yabo Wang, Heming Lin, Hanhua Cheng, Rongjia Zhou

**Affiliations:** Hubei Key Laboratory of Cell Homoeostasis, College of Life Sciences, Wuhan University, Wuhan, 430072 P. R. China

## Abstract

Intracellular membrane trafficking is essential for eukaryotic cell existence. Here, we show that RAB37 activation through GTP binding recruits ATG5-12 to isolation membrane and promotes autophagosome formation through the ATG5-ATG12-ATG16L1 complex. RAB37 is localized on the isolation membrane. It can bind directly with ATG5 and promotes formation of the ATG5-12-16 complex. Mutation analysis reveals that GTP-bound RAB37 exhibits an enhanced interaction with ATG5-12 and GDP-stabilised mutation impairs the interaction. RAB37 promotes ATG5-12 interaction with ATG16L1, thus facilitates lipidation of LC3B in a GTP-dependent manner to enhance autophagy. Notably, ablation of RAB37 expression affects the complex formation and decreases autophagy, whereas forced RAB37 expression promotes autophagy and also suppresses cell proliferation. Our results demonstrate a role of RAB37 in autophagosome formation through a molecular connection of RAB37, ATG5-12, ATG16L1 up to LC3B, suggesting an organiser role of RAB37 during autophagosomal membrane biogenesis. These findings have broad implications for understanding the role of RAB vesicle transport in autophagy and cancer.

## Introduction

Intracellular membrane trafficking between organelles is essential for nearly all eukaryotic cells. The process involves vesicle budding, motility, tethering and fusion with the specific target membrane. The organisation and transport of membrane organelles ensure different molecules or cell parts with different biochemical natures to be sequestered and allow for the exchange of the materials between compartments. As key organisers of these processes, RAB GTPases regulate membrane functions through a switch between two distinct conformations: the GTP-bound “on” and the GDP-bound “off” forms [1]. Membrane trafficking regulation has both physiological and pathological implications. RAB pathway dysfunctions are associated with many human diseases, such as cancer [2], mental retardation [3], Parkinson’s disease [4], immunodeficiency [5] and obesity [6].

The RAB family consists of small GTPases and is a part of the RAS superfamily. At least 60 RAB members have been identified in humans [1]. RAB GTP proteins (active) localized in membranes can recruit specific effector proteins to target membranes, whereas RAB GDP proteins (inactive) disassociate from the membrane to the cytosol and are recycled. A number of RAB proteins are involved in autophagy regulation, especially in the processes of autophagosome formation and autophagosome–lysosome fusion [7]. For example, Ypt1, a homologue of RAB1 in yeast, can interact with Atg11 and is required for autophagosome formation in a GTP-dependent manner [8]. It can also regulate autophagy by recruiting its effector Atg1 [9]. Ypt6 participates proper traffic of Atg9 to the preautophagosomal structure under a high temperature stress [10]. Knockdown of RAB1b and RAB2 increases levels of the key autophagic protein LC3B-II [4]. RAB26 and RAB33B are required for isolation membrane formation by interaction with ATG16L1 [11, 12]. Rab19 can directly bind to Atg16 in a GTP-dependent manner to promote intestinal secretory cell differentiation in *Drosophila* [13]. In addition, some other RAB members are involved in maturation of autophagic vacuoles, such as RAB7, RAB8B and Rab2 (*Drosophila*) [14–17]. Despite these progresses, however, the molecular mechanisms and the spatiotemporal regulations of the crosstalk between GTPases and autophagy remain largely unknown.

RAB37 is localized on various vesicles related to secretion and exocytosis, including secretory granules in bone marrow mast cells [18], dense-core vesicles in PC12 cells [19], insulin secretory granules [20] and Weibel-Palade bodies (endothelial-cell-specific organelles) [21]. RAB37 regulates mast cell degranulation through its effector protein [22], TNF-alpha secretion from activated macrophages [23] and insulin secretion through exocytosis [24]. RAB37 is also associated with endothelial cell function and embryogenesis in zebrafish [25]. In addition, RAB37 is involved in tumour cell growth [26–29]. A recent study indicated that RAB37 can suppress metastasis through the TIMP1-MMP9 pathway [30], highlighting an importance of RAB37 protein in cancer. Despite these research efforts, possible association of RAB37 with autophagy and its regulation mechanisms remain unclear.

In this study, in an attempt to better understand the functions and molecular mechanisms of RAB37 in autophagy, we investigate ATG proteins interacting with RAB37 and find that RAB37 as a key organiser of autophagosome formation promotes autophagy through the ATG5-ATG12-ATG16L1 complex. It binds directly with ATG5, and promotes autophagosome formation in a GTP-dependent manner. RAB37 knockdown can decrease autophagy and increase tumour metastasis in vivo. Our results provide clear evidence and underlying molecular mechanism that RAB37 promotes autophagy. These findings have broad implications for understanding the role of RAB37 vesicle transport in autophagy and cancer.

## Results

### RAB37 directly interacts with ATG5

**Fig. 1:**
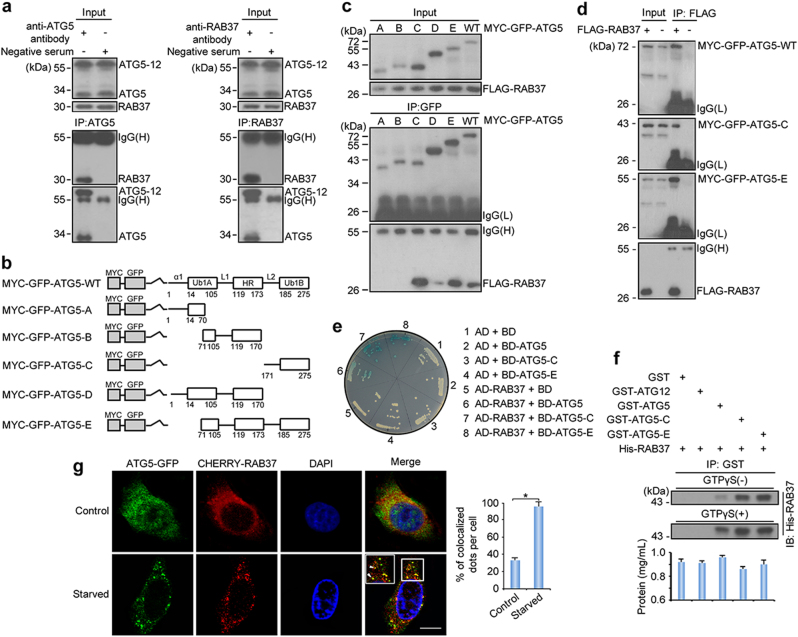
RAB37 directly interacts with ATG5. **a** Co-immunoprecipitation analysis between endogenous RAB37 and ATG5 in 293T cells. The lysates were immunoprecipitated with anti-ATG5, anti-RAB37 antibody or negative serum respectively, followed by immunoblotting with the anti-RAB37 or anti-ATG5 antibody respectively. **b** A schematic diagram of human ATG5 wild type and deletion mutants. The conserved domains (α1, UblA, L1, HR, L2 and Ub1B) are indicated in wild type ATG5. **c**, **d** Interactions of RAB37 with ATG5 mutants. 293T cells were transiently co-transfected with FLAG-RAB37 and MYC-GFP-ATG5 mutants or ATG5 wild type. Cell lysates were examined by Western blotting using an anti-GFP or anti-FLAG antibody. For co-immunoprecipitation assays, the lysates were immunoprecipitated with an anti-GFP antibody followed by immunoblotting with an anti-FLAG or anti-GFP antibody (**c**), or immunoprecipitated with an anti-FLAG antibody followed by immunoblotting with an anti-GFP or anti-FLAG antibody (**d**). **e** Identification of the interaction between RAB37 and ATG5 by yeast two-hybrid assay. Y2HGold cells were transformed with plasmids as indicated. The growth of yeast colonies containing relevant plasmids on SD/-His/-Leu/-Trp medium showed that RAB37 interacted with wild-type ATG5, and the truncated ATG5-C and -E in yeast cells. AD, pGADT7; BD, pGBKT7. **f** In vitro GST-pulldown assay showed interaction of RAB37 with ATG5 and ATG12, which was dependent on GTP binding activity of RAB37. GST-ATG5, GST-ATG5-C, GST-ATG5-E, and GST-ATG12 were incubated with His-tagged RAB37 respectively, when present of GTPγS or not. Proteins pulled down with glutathione-agarose were subjected to SDS-PAGE followed by immunoblotting with an anti-RAB37 antibody. Bottom: total proteins per lane were determined by the Pierce BCA protein assay kit. **g** Co-localization of RAB37 puncta with ATG5. COS-7 cells were co-transfected with CHERRY-RAB37 and ATG5-GFP. Before fixation, the cells were cultured in starvation medium EBSS for 1 h. Co-localization dots were observed between CHERRY-RAB37 and ATG5-GFP as indicated in arrowheads. Square areas in white line were enlarged. Images were captured using confocal microscopy. The nuclei were counterstained with DAPI (blue). Percentage of co-localized dots (CHERRY-RAB37 + ATG5-GFP (yellow) / ATG5-GFP (green)) upon starvation was determined in comparison with non-starvation controls. Data are presented as means ± S.D. * stands for *P* < 0.05 (*n* = 3 independent experiments). Scale bar, 10 µm

To investigate whether RAB37 is involved in autophagy, we detected RAB37 interaction with key autophagy protein ATG5. Co-immunoprecipitation experiments showed that RAB37 was associated with ATG5 and ATG5-12 (Fig. 1a). To map interactive domains of ATG5 with RAB37, we used deletion mapping and reciprocal co-immunoprecipitation analysis, which revealed that the L2-UblB of ATG5 was main domain to interact with RAB37 (Fig. 1b-d). To further explore whether the interaction was direct or indirect, yeast two-hybrid and GST-pulldown assays were used. The L2-UblB domain in the C-terminal of ATG5 showed a direct interaction with RAB37 after test of various deletion variants, while RAB37 did not directly interact with ATG12. Yeast two-hybrid assay indicated the direct binding between RAB37 and ATG5 (Fig. 1e). As GTP-γ-S is a non-hydrolyzable GTP analogue, we used GTPγS assay to investigate whether RAB37 interaction with ATG5 is dependent on its GTP-binding function. GTPγS assay showed that the direct interaction depended on GTP-binding of RAB37 (Fig. 1f and Supplementary Fig. S1). Confocal fluorescence microscopy showed that obvious co-localization of CHERRY-RAB37 and ATG5-GFP puncta were detected when co-transfected upon starvation induction (Fig. 1g). ATG5 truncated mutants (-C and -E) have stronger interactions with RAB37 in comparison with full-length ATG5, which may reflect an appropriate topological structure of ATG5 domain interaction with RAB37. These data suggested that RAB37 directly interacts with ATG5.

### RAB37 interacts with ATG5 in a GTP-dependent manner

**Fig. 2:**
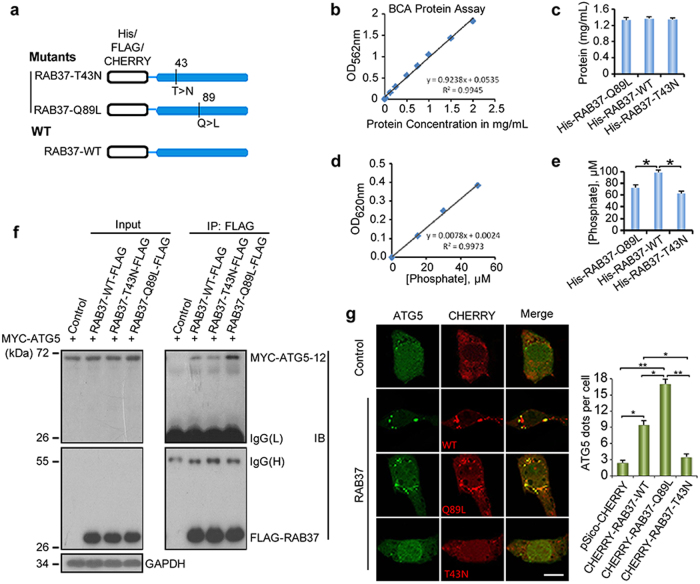
RAB37 interacts with ATG5 in a GTP-dependent manner. **a** A schematic representation of the RAB37 mutants. Inactive RAB37-T43N is a GDP-stabilised mutant, and active RAB37-Q89L is a GTP-stabilised mutant. **b**, **c** Protein quantification was determined by Pierce BCA protein assay kit. Three RAB37 proteins (wild type and two mutants) were purified from *E. coli*. **d**, **e** RAB37 (and its mutants -Q89L and -T43N) GTPase activity assays by Quantichrome ATPase/GTPase assay kit according to a standard curve. **f** RAB37 interacts with ATG5 in a GTP-dependent manner. MYC-ATG5 was transiently co-transfected with FLAG-RAB37-WT, FLAG-RAB37-T43N, FLAG-RAB37-Q89L or control plasmid into 293T cells respectively. For co-immunoprecipitation, the lysates were immunoprecipitated with an anti-FLAG antibody followed by immunoblotting with an anti-MYC and anti-FLAG antibodies. GAPDH was used as an internal control. **g** RAB37 transfection promotes formation of ATG5 positive puncta in a GTP-dependent manner. COS-7 cells transfected with CHERRY-RAB37-WT, CHERRY-RAB37-Q89L, CHERRY-RAB37-T43N or control vector pSico-CHERRY respectively. Left: representative images of cells with ATG5-GFP (green) and CHERRY-RAB37 (its mutants or control vector CHERRY, red). Right: ATG5 positive dots were quantified from ~20 cells. Data are presented as means ± S.D. * stands for *P* < 0.05, ** stands for *P* < 0.01 (*n* = 3 independent experiments)

To test whether RAB37 functions as a GTPase, we used two RAB37 mutants, a GTP-hydrolysis-deficient, constitutively active mutant (RAB37-Q89L) and a GDP-stabilised, constitutively negative mutant (RAB37-T43N) (Fig. 2a), and tested effects of ATG5 interaction with the RAB37 mutants. As RAB37 is a novel GTPase, we first detected the GTPase activity of RAB37 and its mutants. Protein quantification was determined by Pierce BCA protein assay in these GTPase activity experiments (Fig. 2b, c). RAB37 wild type showed an obvious dephosphorylation from GTP, in comparison with two RAB37 mutants (RAB37-Q89L had a constantly binding with GTP and RAB37-T43N did not bind with GTP) (Fig. 2d, e). Co-immunoprecipitation experiments showed that the RAB37-Q89L mutant had an obvious interaction with ATG5 upon starvation induction, whereas the RAB37-T43N mutant was scarcely associated with ATG5 (Fig. 2f). Confocal fluorescence microscopy showed that RAB37 transfection promotes formation of ATG5 positive puncta in a GTP-dependent manner (Fig. 2g). Together, these data suggested that RAB37 interacts with ATG5 in a GTP-dependent manner.

### RAB37 localization on autophagosome in a GTP-dependent manner

**Fig. 3:**
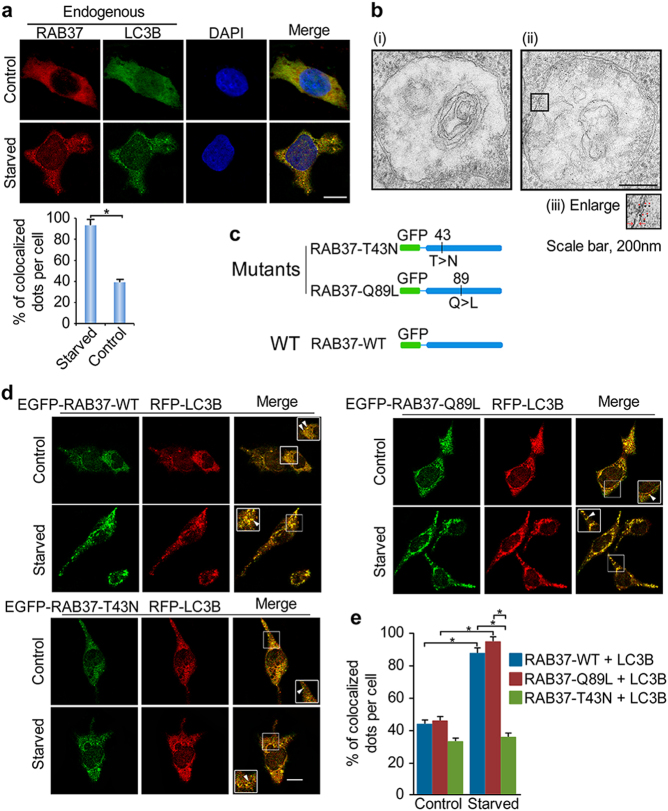
RAB37 co-localization with LC3B puncta upon starvation induction. **a** Puncta co-localization of endogenous RAB37 with LC3B upon starvation induction. COS-7 cells were cultured in normal or starvation medium EBSS for 1 h. Percentage of co-localized dots (RAB37 + LC3B (yellow) / LC3B (green)) upon starvation was quantified in comparison with non-starvation controls. Data are presented as means ± S.D. * stands for *P* < 0.05 (*n* = 3 independent experiments). Scale bar, 10 µm. **b** Localization of RAB37 puncta on autophagosome revealed by immuno-EM. HeLa cells were transiently transfected with pCMV-Tag2B or FLAG-RAB37, respectively. The cells were cultured in starvation medium EBSS for 1 h and examined using anti-FLAG antibody and then anti-mouse IgG conjugated to 6 nm gold particles. (i) Negative control, (ii) representative image of the localization of RAB37 puncta on autophagosome, and square areas in black line were enlarged and showed in (iii). Red triangles, gold particles; red arrowheads, the double membranes of autophagosome; Scale bar, 200 nm. **c** A schematic representation of the RAB37 mutants used in (**d**). RAB37-T43N is a GDP-stabilised mutant, and RAB37-Q89L is a GTP-stabilised mutant. **d** RAB37-Q89L promotes its co-localization with LC3B upon starvation induction. COS-7 cells were transiently co-transfected with RFP-LC3B and GFP-RAB37-T43N, -RAB37-Q89L or -WT respectively. The cells were cultured in starvation medium EBSS for 1 h and then examined using confocal microscopy. Non-starvation treatment was used as a control. Co-localizing structures are indicated in arrowheads. Square areas in the inset were enlarged. **e** Counting of co-localized puncta between RAB37 and LC3B per cell. Percentages of co-localized dots (RFP-LC3B + GFP-RAB37-WT, -Q89L or -T43N (yellow) / RFP-LC3B (red)) upon starvation were determined in comparison with non-starvation treatments. The co-localized dots were counted from ~30 cells. * stands for *P* < 0.05

To further investigate the RAB37 functions in autophagy, we explored whether RAB37 localizes on autophagosome upon starvation induction. Immunofluorescence and confocal fluorescence microscopy analyses showed co-localization of endogenous RAB37 with LC3B puncta upon starvation induction (Fig. 3a). Localization of RAB37 on autophagosome, mainly around its membrane, was further determined by immuno-EM analysis (Fig. 3b). Co-localization of RAB37-Q89L or RAB37-wild type with LC3B were further observed, whereas a marked reduction of co-localization was detected when the RAB37-T43N mutant was co-transfected (Fig. 3c-e). These results indicated that RAB37 localization on autophagosome is in a GTP-dependent manner.

### RAB37 promotes formation of the ATG5-12-16L1 complex in a GTP-dependent manner

**Fig. 4:**
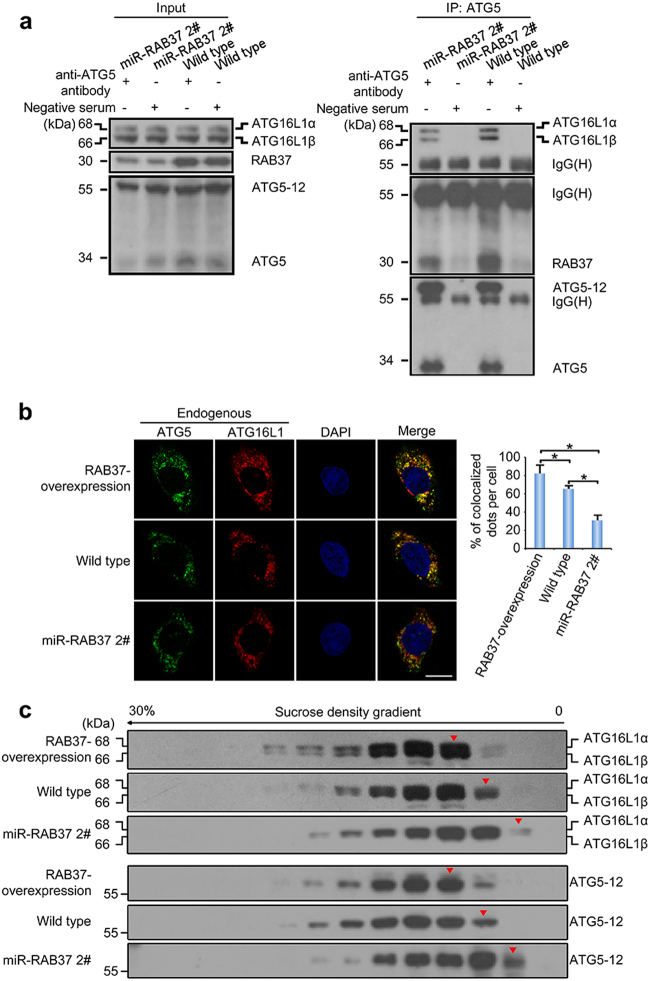
RAB37 knockdown interferes with formation of ATG12-ATG5-ATG16L1 complex. **a** ATG5 interacts with RAB37 and ATG16L1 respectively. Two cell lines (RAB37 knockdown and control HeLa cells) were cultured and then starved for 1 h before harvest. The lysates were immunoprecipitated with anti-ATG5 antibody or negative serum, followed by immunoblotting with the anti-RAB37, anti-ATG16L1 or anti-ATG5 antibody. **b** RAB37 knockdown interferes with co-localization of endogenous ATG5 and ATG16L1 proteins. RAB37 overexpression, RAB37 knockdown and wild type HeLa cells were starved in the medium EBSS for 1 h before stained by immuno fluorescence with anti-ATG5, anti-ATG16L1 antibodies and then anti-mouse IgG FITC conjugated antibody (green), or anti-rabbit IgG TRITC conjugated antibody (red) respectively. The nuclei were counterstained with DAPI (blue). Confocal images were taken. Scale bar, 10 µm. Percentages of co-localized dots (ATG5 + ATG16L1 (yellow)/ATG5 (green)) in three HeLa cell lines were determined and the co-localized dots between ATG5 and ATG16L1 were counted from ~15 cells respectively. * stands for *P* < 0.05. **c** RAB37 knockdown affects formation of ATG12-ATG5-ATG16L1 complex. Lysates of three cell lines (RAB37 overexpression, RAB37 knockdown and wild type HeLa cells) were separated using continuous sucrose gradients (0–30%). Fractions were subjected to Western blotting using the indicated antibodies. Red triangles indicate the size shift of the complex

To test a possible role of RAB37 in formation of the ATG5-12-16L1 complex, we established RAB37 knockdown HeLa cell lines (Supplementary Fig. S2a). Co-immunoprecipitation experiments showed that ATG5 interacted with RAB37 and ATG16L1 respectively, and RAB37 knockdown inhibited interaction of the ATG5-12 with ATG16L1 (Fig. 4a). Immunofluorescence analysis of endogenous ATG5 and ATG16L1 in the cells also revealed that RAB37 knockdown inhibited ATG5 co-localization with ATG16L1, while RAB37 forced expression promoted the co-localization (Fig. 4b). We further investigated effect of RAB37 on formation of the ATG5-12-16L1 complex. Sucrose density gradient centrifugation of the cell lysates and Western blot analysis showed that knockdown of RAB37 decreased molecular weight of the complex ATG5-12-16L1, while RAB37 overexpression promoted the complex formation (Fig. 4c). These data suggested a role of RAB37 in formation of the ATG5-12-16L1 complex.

**Fig. 5:**
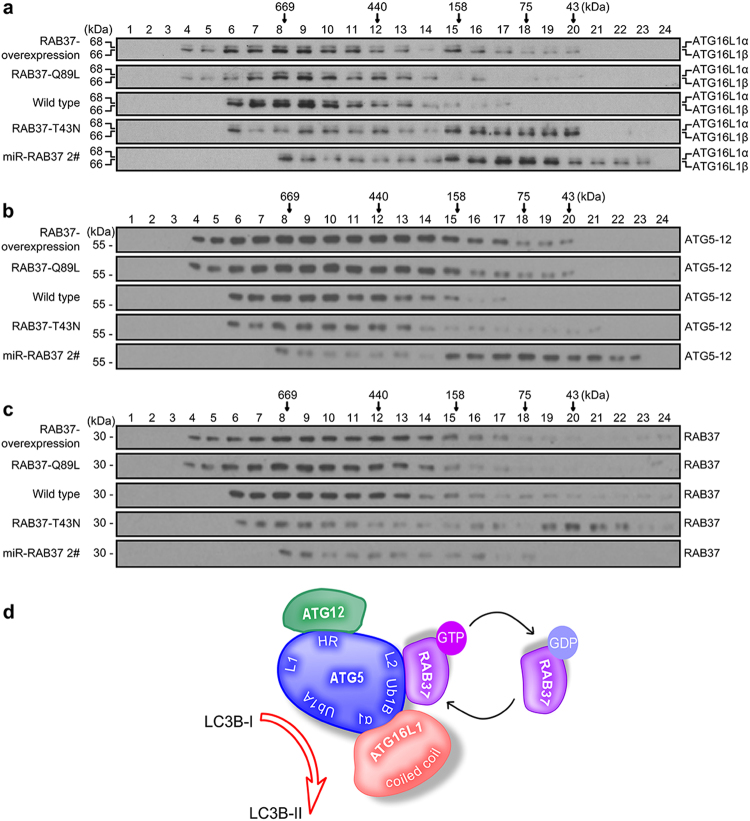
RAB37 promotes formation of the ATG12-ATG5-ATG16L1 complex in a GTP-dependent manner. **a**–**c** Gel-filtration analysis of the ATG12-ATG5-ATG16L1 complex by chromatography. Cell lysates of three cell lines (RAB37 overexpression, RAB37 knockdown and control HeLa cells) and two transiently transfected cells (FLAG-RAB37-Q89L and FLAG-RAB37-T43N) were separated by size-exclusion chromatography. Fractions were subjected to Western blotting with anti-ATG16L1 (**a**), anti-ATG5 (**b**) or anti-RAB37 antibody (**c**), respectively. The positions of the molecular mass standards in chromatography are showed in arrows. **d** Work model of RAB37 function together with ATG5-12 and ATG16L1 for autophagy

Gel filtration was used further to determine the role of RAB37 in ATG12-ATG5-ATG16L1 complex assembly. RAB37 knockdown interfered with formation of ~800 kDa multimeric ATG protein complex including eight sets of ATG5-12-16L1, even lead to monomer ATG5-12 and ATG16L1 (Fig. 5a-c). We also investigated whether the complex formation is dependent on GTP-binding of RAB37. The GTP-hydrolysis-deficient, constitutively active mutant (RAB37-Q89L) can increase molecular size of the complex ATG5-12-16L1, while constitutively negative mutant (RAB37-T43N) affected the complex formation (Fig. 5a-c). These data indicated that RAB37 promotes formation of the ATG5-12-16L1 complex in a GTP-dependent manner, thus facilitates lipidation of LC3B at initiation of autophagosome formation (Fig. 5d).

### RAB37 puncta are co-localized with isolation membranes in an ATG5 dependent manner

**Fig. 6:**
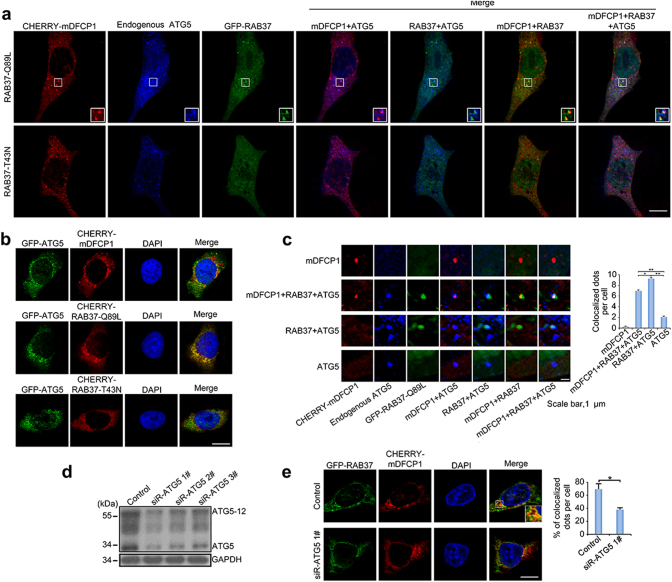
RAB37 puncta are co-localized with isolation membranes. **a** Co-localization of RAB37 puncta with ATG5 and an omegasome marker DFCP1. COS-7 cells were co-transfected with RAB37 mutants (GFP-RAB37-Q89L and -RAB37-T43N) and CHERRY-mDFCP1. The cells were starved in the medium EBSS for 1 h befor harvest. Endogenous ATG5 protein was stained by immune fluorescence using anti-ATG5 antibody and then anti-rabbit IgG AMCA conjugated antibody (blue). Confocal images were taken. Scale bar, 10 µm. The outlined region is magnified in the inset. **b** ATG5 co-localizations with RAB37-Q89L, RAB37-T43N and DFCP1, respectively. GFP-ATG5 was co-transfected with CHERRY-RAB37-Q89L, CHERRY-RAB37-T43N and CHERRY-DFCP1 in HeLa cells, respectively. Scale bar, 10 µm. **c** Representative images of DFCP1-positive, RAB37-Q89L/DFCP1/ATG5-positive, RAB37-Q89L/ATG5-positive and ATG5-positive puncta in COS-7 cells from (**a**). Positive dots were quantified from ~20 cells. Scale bar, 1 µm. Data are presented as means ± S.D. * stands for *P* < 0.05, ** stands for *P* < 0.01 (*n* = 3 independent experiments). **d** Determination of effective ATG5 siRNA. Three siRNAs and a negative control siRNA were transfected into HeLa cells, and ATG5 and ATG5-12 protein levels were analysed by Western blots. **e** ATG5 interference inhibits RAB37 co-localization with CHERRY-mDFCP1. HeLa cells were co-transfected with siRNA (siR-ATG5 1# or negative control), GFP-RAB37 and CHERRY-mDFCP1. The cells were cultured in DMEM with 10% FBS and starved in starvation medium EBSS for 1 h. The images were taken under confocal microscopy. Scale bar, 10 µm. Percentage of co-localized dots (GFP-RAB37 + CHERRY-mDFCP1 (yellow) / CHERRY-mDFCP1 (red)) in controls were determined in comparison with ATG5 knockdown treatments. The co-localized dots were counted from ~20 cells. * stands for *P < *0.05

To confirm the association of RAB37 puncta with isolation membranes, we used RAB37-Q89L and RAB37-T43N mutants to analyse co-localization of RAB37 puncta with ATG5 and an omegasome marker DFCP1 (Double FYVE-containing protein 1, a PtdIns(3)P-binding ER protein). Confocal images showed that RAB37-Q89L puncta were co-localized with DFCP1 and ATG5 upon starvation induction, whereas the co-localization was markedly reduced when RAB37 was functionally mutated in constitutively negative mutation (Fig. 6a, b). Notably, most RAB37/ATG5 positive structures were DFCP1 positive (Fig. 6c), indicating that RAB37 is co-localized with isolation membranes. We further investigate whether ATG5 interference influences the RAB37 co-localization with DFCP1. Confocal fluorescence microscopy analyses showed that ATG5 knockdown significantly prevented the RAB37 co-localization with DFCP1 (Fig. 6d, e). The results suggested that RAB37 puncta are co-localized with isolation membranes in an ATG5 dependent manner.

### RAB37-associated autophagy flux

**Fig. 7:**
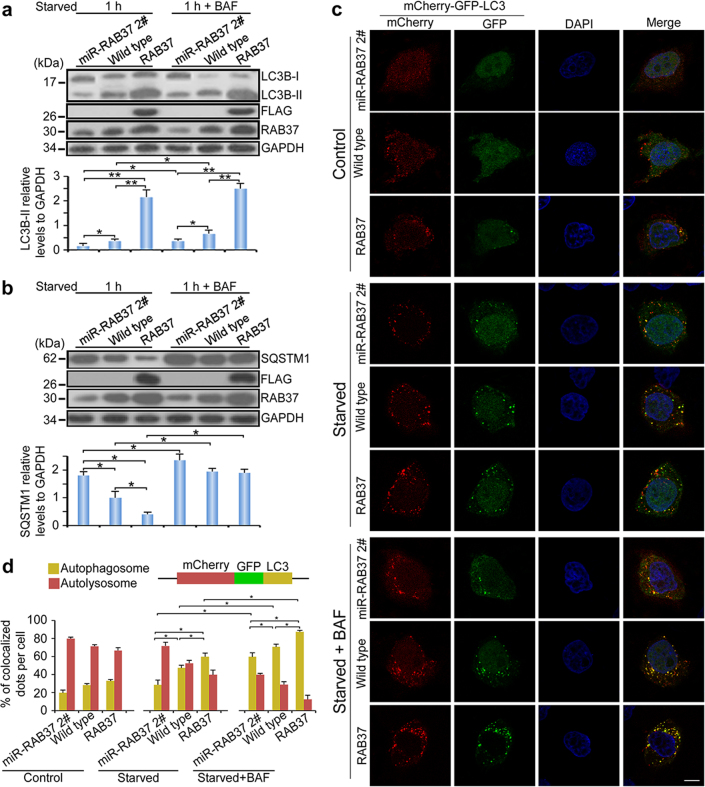
RAB37-associated autophagy flux. **a**, **b** RAB37 knockdown inhibits and over-expression promotes LC3B-II formation. Bafilomycin A_1_ treatment results in protein accumulation of LC3B-II and SQSTM1. RAB37 overexpression, RAB37 knockdown and control HeLa cells were cultured and then starved in EBSS with or without bafilomycin A_1_ (100 nM) for 1 h. The cell lysates were analysed by immunoblotting with antibodies as indicated. GAPDH was used as an endogenous control. Bottom: western blots were quantified for LC3B-II /GAPDH ratio and the SQSTM1/GAPDH ratio. Data are presented as means ± S.D. * stands for *P* < 0.05, ** stands for *P* < 0.01 (*n* = 3 independent experiments). **c** Autophagy flux revealed by the mCherry-GFP-LC3 tandem reporter in RAB37 knockdown (miR-RAB37 2#), over-expressed (stably expressing RAB37) and wild-type HeLa cell lines. Representative images of the cells transfected with the mCherry-GFP-LC3 reporter and grown in normal (control), EBSS medium (1 h) or EBSS with bafilomycin A_1_ (100 nM) addition (1 h) respectively. Single channel (red, green or blue) and merged images were taken by confocal microscopy. **d** Statistic analysis of vesicles positive for both GFP and mCherry (autophagosomes) and for mCherry (autolysosomes) (>15 cells per experiment) in (**c**). Percentages of co-localized dots (mCherry-GFP-LC3 (yellow) / mCherry -LC3 (red)) were counted. Data are presented as means ± S.D. * stands for *P* < 0.05, (*n* = 3 independent experiments)

To investigate RAB37-associated autophagy process, we tested the RAB37-involved autophagy flux through RAB37 RNAi and forced expression. Western blot analysis showed that RAB37 knockdown inhibited LC3B-II formation, whereas overexpression promoted the formation in the cell line upon starvation induction, and LC3B-II was accumulated during bafilomycin A_1_ treatment (Fig. 7a). In addition, autophagic substrate SQSTM1 level had a corresponding change with LC3B-II (Fig. 7b). Autophagy flux tests were further performed using a tandem fluorescent indicator, mCherry-GFP-LC3, in RAB37 stable overexpression and knockdown cell lines. Since green fluorescence of the fusion protein is very sensitive to the acidic environment of lysosomes and quickly quenched in autolysosomes, just red fluorescence could be detected in the autolysosomes [31, 32]. Fluorescence analysis using the tandem fluorescent indicator system in these cell lines showed that RAB37 knockdown significantly inhibited formation of autophagosomes, while overexpression promotes the formation. Further bafilomycin A_1_ treatment showed a significant accumulation of autophagosomes in RAB37 overexpression (Fig. 7c, d). Together, these data demonstrate that RAB37 promotes formation of autophagosomes, but does not affect its degradation process through lysosomes, upon starvation induction.

### RAB37 promotes autophagy in vivo

**Fig. 8:**
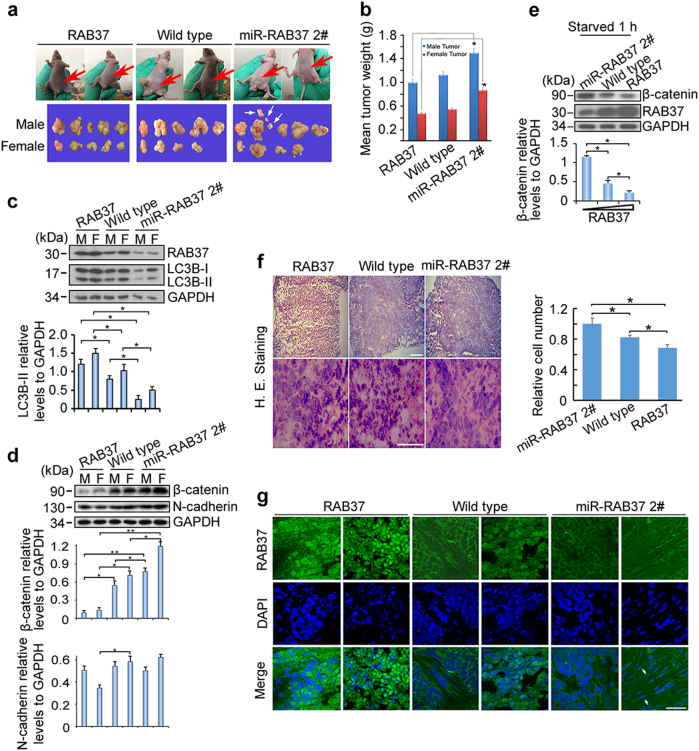
RAB37 promotes autophagy in vivo. **a** Nude mouse experiments. Three cell lines (HeLa cells stably expressing RAB37, miR-RAB37 2# and control cells) were used to implant nude mice. After 4 weeks, the tumours from both males and females were collected. Red arrows show tumour position in mouse. White arrows indicate tumour migration to other places in some mice. **b** RAB37 knockdown promotes tumour growth. Each bar represents the mean, and the error bars indicate the standard deviation. * stands for *P* < 0.05. **c**, **d** Tumour samples from three types of nude mice were examined through immunoblotting with anti-LC3B and anti-RAB37 (**c**), anti-β-catenin and N-cadherin antibodies (**d**). Both β-catenin and N-cadherin are EMT markers. GAPDH was used as an endogenous control. The molecular marker is showed on the left. Bottom: western blots were quantified for the LC3B-II/GAPDH, β-catenin/GAPDH, N-cadherin/GAPDH ratios. Data are presented as means ± S.D. * stands for *P* < 0.05, ** stands for *P* < 0.01 (*n* = 3 independent experiments). **e** RAB37 interference increases β-catenin protein level in HeLa cell culture in vitro. Bottom: western blots were quantified for β-catenin/GAPDH ratio. **f** Haematoxylin and eosin staining of three tumour types. Scale bar, 200 nm (up panels) or 100 nm (down panels). Relative cell numbers in one image was quantified. Data are presented as means ± S.D. * stands for *P* < 0.05, (*n* = 3 independent experiments). **g** Immunofluorescence analysis of tumour samples. Arrows show a longer tumour cell morphologically and a denser cell growth in RAB37-knockdown. The tumour sections were immunostained with an anti-RAB37 antibody (FITC). The nuclei were counterstained with DAPI (blue). The images were captured using confocal microscopy. Scale bar, 50 nm

To further investigate the autophagy regulation by RAB37, the stable overexpression and knockdown cell lines were used to implant nude mice. Because autophagy suppression can promote tumorigenesis, [33] we further examined a role for RAB37 in autophagy regulation through tumorigenesis in vivo. Cell counting, scratch migration and soft agar for colony formation assays showed that RAB37 knockdown promoted cell migration and proliferation, whereas RAB37 overexpression had an inhibitory effect (Supplementary Fig. S2b-e). The cells stably expressing RAB37 and miR-RAB37 2# were further used to implant nude mice. After 4 weeks, tumours from both males and females were collected and weighed (Fig. 8a). RAB37 knockdown promoted tumour growth, whereas RAB37 overexpression inhibited tumour growth. Notably, some tumours, when RAB37 knocked down, migrated to other body regions, such as the leg and the back, which indicated that RAB37 deprivation resulted in tumour metastasis. The difference in tumour growth between these two groups was significant (Fig. 8b). Interestingly, the tumour size in females was significantly smaller than in males, indicating a sex-associated regulation of RAB37 in tumour growth. The tumour samples from nude mice were then examined through immunoblotting with anti-LC3B and anti-RAB37 antibodies, which showed that LC3B-II levels decreased in RAB37 knocked-down tumours, whereas LC3B-II levels increased in RAB37-overexpressed tumours (Fig. 8c). Further epithelial-mesenchymal transition (EMT) marker tests showed that β-catenin and N-cadherin were increased in RAB37-knockdown tumours, whereas it was decreased in RAB37-overexpressed tumours (Fig. 8d). Meanwhile, the increase of β-catenin was also obvious in RAB37-knockdown cell cultures (Fig. 8e). Histological and immunofluorescence analysis of tumour samples showed a denser cell growth in RAB37-knockdown than in RAB37-overexpressed tumours (Fig. 8f, g), a phenotype similar to the EMT phenotype. Furthermore, immunofluorescence analysis of clinical tumours samples in humans showed markedly decreased RAB37 expression in prostate adenocarcinomas, prostate transitional cell carcinomas, stomach adenocarcinoma and cervical squamous cell carcinomas (Supplementary Fig. S3a-c). In addition, RAB37 protein was detected in abnormal nuclei in some cervical squamous cell carcinomas (Supplementary Fig. S3c). Taken together, these results suggested that RAB37 promotes autophagy, thus at least partly prevents tumour metastasis.

## Discussion

Collectively, we provide direct evidence that RAB37 is linked to autophagy and underlying molecular mechanism. RAB37 is located at the isolation membrane upon autophagy initiation. It interacts directly with ATG5, a key protein at the initiation of pre-autophagosome formation, which recruits ATG16L1 to pre-autophagosomal membranes to form ATG5-ATG12-ATG16L1 complex. Subsequently the complex promotes lipidation of LC3B through a conjugation of phosphatidylethanolamine, thus generating the crucial molecule, LC3B-II, to facilitate the nascent autophagosome genesis. The process is associated with the transition between the active RAB37 GTP form and the inactive RAB37 GDP form. Nevertheless, RAB37 does not affect degradation process of autophagosomes through lysosomes as revealed by RAB37-associated autophagy flux analysis. Thus, RAB37 is a key organiser of autophagosomal membrane biogenesis at the beginning of autophagy.

Over 60 RAB members together with their distinct effectors endow RAB proteins with diversity of functions in vesicle transportation within cells, such as vesicle formation, movement, fusion with other membrane and determining compartment specificity and organelle identity. For example, Rab19 can promote intestinal secretory cell differentiation via regulation of Slit/Robo signalling in *Drosophila* [13]. RAB33A, an isoform of RAB33 family, participates in regulation of amylase release from parotid acinar cells [34]. RAB33A has also a role in axon outgrowth by mediating anterograde vesicular transport for membrane exocytosis and their concomitant fusion at the growth cones [35]. Several other RAB proteins are involved in autophagy processes [7], such as Ypt1/RAB1 [9], Rab2 [16, 17], RAB7 [36], RAB26 [12] and RAB33 [37], including autophagosome formation and fusion with lysosome at the late stage autophagy. RAB33A/B can interact with ATG16L1 [11] and RAB37 interacts with ATG5 instead of ATG16L1, which indicate that RAB33 and RAB37 are involved in distinct processes in autophagosome formation, probably in different biological activities in particular cell types [38, 39]. RAB37 exerts its function through ATG12-ATG5-ATG16L1 to regulate autophagosome biogenesis in a temporospatial manner. The regulation is dependent on RAB37 ability of GTPases to cycle regularly between GTP- and GDP-bound states, in which RAB37 may act as a timer for on/off regulatory function at the initiation stage of autophagosome formation [40].

Because autophagy suppression can promote tumorigenesis [33], an intriguing question is whether RAB37 is involved in tumour suppression by promoting autophagy. This report answers the interesting question. RAB37-knockdown tumours showed tumour migration and an EMT phenomenon in both histological characteristics and EMT markers, indicating a regulatory role of RAB37 in tumour metastasis. These results are consistent with the observation that in certain clinical tumour samples in humans with decreased RAB37 expression, the protein is often translocated into abnormal nuclei. Tumourigenesis is closely associated with autophagy [33]. Some proteins regulate both autophagy and tumorigenesis. For example, ATG6 (BECLIN1) phosphorylation participates in autophagy inhibition and oncogenesis [41]. Previous study showed that RAB37 can suppress metastasis through the TIMP1-MMP9 pathway [30]. RAB37 may exert its tumour suppression role through other processes, such as epigenetic modification [26], in addition to promoting autophagy. As a key organiser of vesicle transport, RAB37 may function in tumorigenesis through exchange of the materials between compartments. Indeed, RAB37 plays a role in exocytosis and secretion [23, 24]. These results indicate that RAB37 may function in multiple molecular processes in regulations of tumour metastasis. RAB37-ATG5 pathway linking to autophagosome formation highlights an importance of intracellular membrane trafficking involving RAB vesicles in maintenance of membrane homoeostasis.

Based on these findings, we propose a model to depict a role for RAB37 in autophagosome formation (Fig. 5d). The active RAB37-GTP interacts directly with ATG5, and promotes formation of ~800 kDa multimeric ATG protein complex including eight sets of ATG5-12-16L1. Subsequently, the ATG5-12-16 complex recruits and lipidates LC3B-I to form active LC3B-II, which accelerates autophagosomal formation. RAB37-GTP is then transformed into the inactive RAB37-GDP through the GTPase accelerating protein. Afterwards, RAB37-GDP disassociates from the vesicle membrane and spreads to the cytosol for recycling to organise another initiation process of autophagosomal formation when autophagy induction. Our findings demonstrate a molecular link of RAB37-ATG5-12-16-LC3B-II to autophagosome formation. The molecular mechanism has broad physiological and pathological implications for understanding the role of RAB37 in autophagy promotion, and in particular, cancer prevention and early detection.

## Materials and methods

### Antibodies and reagents

Primary antibodies: Anti-ATG5 (Cat# 10181-2-AP) was purchased from Proteintech Group, Rosemont, IL, USA. Anti-ATG5 (Cat# ET1611-38) was purchased from HuaAn Biotechnology, HangZhou, China. Anti-ATG5 (Cat# sc-133158) was purchased from Santa Cruz Biotechnology, Dallas, Texas, USA. Anti-ATG16L1 (Cat# 8089) and anti-LC3B (Cat# 3868) were purchased from Cell Signalling Technology, Danvers, MA, USA. The anti-GAPDH antibody (Cat# CW0100A) was from CWBIO, Beijing, China. The anti-β-catenin (Cat# 51067-2-AP), anti-N-cadherin (Cat# 22018-1-AP) and anti-p62 (SQSTM1) (Cat# 18420-1-AP) antibodies were from Proteintech Group, Rosemont, IL, USA. The anti-FLAG antibody (Cat# F3165) and monoclonal anti-LC3B (Cat# SAB4200361, produced in mouse) were from Sigma-Aldrich, St Louis, MO, USA. The anti-MYC (Cat# 11667149001) and anti-GFP antibodies (Cat# 11814460001) were from Roche Applied Science, Indianapolis, IN, USA. The anti-RAB37 antibody was produced by Beijing Protein Institute (Beijing, China) against the peptide C*EPSFQIRDYVESQ in rabbits or purchased from Proteintech Group, Rosemont, IL, USA (Cat# 13051-1-AP).

Secondary antibodies: peroxidase-conjugated AffiniPure goat anti-Mouse IgG, light chain specific (Cat# 115-035-174), peroxidase-conjugated AffiniPure F(ab′)2 fragment rabbit anti-mouse IgG, Fc_γ_ fragment specific (Cat# 315-036-046), and peroxidase-conjugated AffiniPure goat anti-rabbit IgG, Fc fragment specific (Cat# 111-035-008) were purchased from Jackson ImmunoResearch Laboratories, West Grove, PA, USA. Goat anti-rabbit IgG, (H + L), horseradish peroxidase conjugated antibody (Cat# 31460), and goat anti-mouse IgG, (H + L), horseradish peroxidase conjugated antibody (Cat# 31430) were from Pierce Biotechnology Company, Rockford, IL, USA.

Fluorescein antibodies: FITC-conjugated ImmunoPure goat anti-mouse IgG(H + L) (Cat# ZF-0312), FITC-conjugated ImmunoPure goat anti-rabbit IgG(H + L) (Cat# ZF-0311) and TRITC-conjugated ImmunoPure goat anti-rabbit IgG(H + L) (Cat# ZF-0316) were purchased from Feiyi Technology, Wuhan, Hubei Province, China. AMCA-conjugated AffiniPure goat anti-rabbit IgG (H + L) (Cat# CW0157) was from CWBIO, Beijing, China.

Reagent materials: DAPI (Cat# C1002), G418 (Cat# ST081), Hematoxylin Staining Solution (Cat# C0107) and Eosin Staining Solution (Cat# C0109) were purchased from Beyotime Institute of Biotechnology (Jiangsu Province, China). Blasticidine S hydrochloride (Cat# 15205), bafilomycin A_1_ (Baf A_1_, Cat# B1793) and GTPγS (Cat# G8634) were from Sigma-Aldrich (St. Louis, MO, USA). Tissue and tumour sections (Cat# MC541, Cat# MBN481) were purchased from US Biomax (Rockville, MD, USA). X-α-gal (Cas# 107021-38-5) was purchased from Gold Biotechnology (St. Louis, MO, USA). Aureobasidin A (Cat# 630466), SD/-Trp supplement (Cat# 630413), SD/-Leu supplement (Cat# 630414), SD/-Leu/-Trp supplement (Cat# 630417), SD/-His/-Leu/-Trp (Cat# 630419) supplement were purchased from Clontech Laboratories, Inc (Takara Bio Company, Kyoto, Japan).

### Plasmid constructs

Full-length RAB37 (NM_175738.4) was cloned into pCMV-Tag2B, pSico-Cherry-Flag and pET32a using *Eco*RI and *Xho*I to generate FLAG-RAB37, CHERRY-RAB37 and His-RAB37 respectively. Full-length RAB37 was cloned into pEGFP-C1 using *Xho*I and *Eco*RI to generate GFP-RAB37. Full-length RAB37 was cloned into pGADT7 using *Nde*I and *Bam*HI to generate AD-RAB37. FLAG-RAB37, GFP-RAB37, His-RAB37, and CHERRY-RAB37 were used as vector backbone to generate RAB37 mutants (T43N and Q89L) using the primers [18]:

T43N5′: 5′-CGTCGGCAAAAACTGTTTCCTGATCC-3′; T43N3′: 5′-GGATCAGGAAACAGTTTTTGCCGACG-3′; Q89L5′: 5′-TGCAGGACTGGAGCGCTTCCGCAGTGTGA-3′; and Q89L3′: 5′-AAGCGCTCCAGTCCTGCAGTGTCCCAG-3′.

RAB37-specific (miR-RAB37) or control (miR-lacZ) miRNA target sequences were synthesised and cloned into pcDNATM6.2-GW/Em-GFP-miR containing EGFP and a blasticidin resistance cassette (Invitrogen, Carlsbad, CA, USA). The target sequences for miRNA RAB37 or LacZ were as follows:

miR-RAB37 1#: 5′-AGCGTCACCCATGCTTATTAC-3′; miR-RAB37 2#: 5′-GAAAGAGTGATCCGTTCCGAA-3′; and miR-LacZ: 5′-GACTACACAAATCAGCGATTT-3′.

Full-length ATG5 (NM_004849.3) was cloned into pcDNA3.0-cMyc using *Bam*HI and *Xho*I to generate MYC-ATG5. Full-length ATG5 was cloned into pEGFP-N1 and pSico-MYC-GFP using *Xho*I and *Bam*HI to generate ATG5-GFP and MYC-GFP-ATG5. MYC-GFP-ATG5 was used as a vector backbone to generate ATG5 mutants (MYC-GFP-ATG5-A, MYC-GFP-ATG5-B, MYC-GFP-ATG5-C, MYC-GFP-ATG5-D and MYC-GFP-ATG5-E) using *Xho*I and *Bam*HI. ATG5 and its mutants were cloned into pGEX-4T-1-GST using *Bam*HI and *Xho*I to generate GST-ATG5, GST-ATG5-C and GST-ATG5-E, and were cloned into pGBKT7 using *Nde*I and *Bam*HI to generate BD-ATG5, BD-ATG5-C and BD-ATG5-E. Full-length ATG12 (NM_004707.3) was cloned into pGEX-4T-1-GST using *Bam*HI and *Xho*I to generate GST-ATG12. Full-length LC3B (NM_022818.4) was cloned into pLove-Red-C1 using *Bam*HI and *Xho*I to generate RFP-LC3B. Full-length mDFCP1 (NM_021260.3) was cloned into pSico-Cherry-Flag using *Eco*RI and *Xho*I to generate CHERRY-mDFCP1.

### Cell culture

293T, COS-7 and HeLa cells were cultured in DMEM (Cat# SH30022.01B, HyClone, Logan, USA) with 10% FBS (Cat# P30-330250, PAN-Biotech, Aidenbach, Germany). To establish a stable RAB37-overexpression cell line, HeLa cells were transfected with the FLAG-RAB37 plasmid using FuGENE HD Transfection Reagent (Cat# 04709705001, Roche Diagnostics, Indianapolis, IN, USA). Stably expressing cells were screened using G418 at a final concentration of 800 µg/mL for 4 weeks. To establish stable RAB37 knockdown cell lines, HeLa cells were transfected with miR-RAB37 1# and miR-RAB37 2# plasmids using the FuGENE HD Transfection Reagent. Stably expressing cells were screened using blasticidin at a final concentration of 20 µg/mL for 4 weeks. For starvation treatments, the cells were cultured in EBSS (Cat# SH30029.02, HyClone, Logan, USA) for various times.

### Western blot analyses

Western blotting was performed according to routine protocols. The proteins were extracted from 293T cells, HeLa cells or tumours. The whole extracts were analysed using SDS-PAGE and transferred to a 0.45 µm PVDF membrane (Cat# NK0414, Roche Diagnostics, Indianapolis, IN, USA). The membranes were blocked with 5% non-fat dried milk in TBST (20 mM Tris-HCl, pH 7.5, 150 mM NaCl, 0.1% Tween-20) and incubated with the antibodies overnight at 4 °C, followed by the horseradish peroxidase-conjugated secondary antibody. The protein bands were visualised by incubating membranes with the ECL Plus detecting reagents (Millipore, Billerica, MA, USA). Band intensity values were obtained using ImageJ (version J2, NIH, Maryland, US). Data were prepared as excel files and analysed by microsoft excel software.

### Co-immunoprecipitation assays

To analyse protein interactions in vivo, co-immunoprecipitation assays were performed in 293T and HeLa cells. The cells were starved using EBSS for 1 h before harvest. Cells were lysed in NETN buffer consisting of 50 mM Tris-HCl at pH 8.0, 0.15 M NaCl, 1 mM EDTA, 0.5% NP-40 and a 1x protease inhibitor cocktail (Cat# 04693159001, Roche Applied Science). The cell lysates were incubated with the specified antibody and Protein G Agarose (Cat# 11243233001, Roche) overnight at 4 °C. The resins were collected by centrifugation and then washed five times with NETN buffer. Bound proteins were eluted using loading buffer (50 mM Tris-HCl, 2% SDS, 1% mercaptoethanol, 10% glycerol, 0.1% bromophenol blue, pH 6.8) and separated using 15% SDS-PAGE, followed by immunoblotting with the appropriate antibodies.

### Protein purification, protein concentration detection and GTPase assays

BL21 cells were transformed with His-RAB37-wild type, -Q89L or -T43N respectively. Cells were cultured at 37 °C with 220 rpm and induced with 0.1 mM IPTG at 16 °C for 24 h. Supernatants were filtered using 0.22 µm filters, loaded onto Ni NTA Beads 6FF (Cat# P2010, Solarbio, Beijing, China), and then gradiently eluted by five different densities of imidazole (2 µM, 50 µM, 100 µM, 200 µM, 500 µM). Pooled supernatants were dialyzed against lysis buffer (0.1 M Tris/HCl, pH 7.0-8.0) to remove impurity and then concentrated by polyethylene glycol before use.

The amounts of recombinant proteins were determined by the Pierce BCA protein assay kit (Cat# 23227, Thermo Scientific, Rockford, IL, USA) according to the manufacturer’s protocol. RAB37 GTPase activity was performed using the QuantiChromTM ATPase/GTPase kit (Cat# DATG-200, BioAssay Systems, Hayward, CA, USA) according to the manufacturer’s protocol.

### Sucrose density gradient centrifugation

Three cell lines (RAB37 overexpression, RAB37 knockdown and control HeLa cells) were cultured in DMEM medium with 10% FBS and starved in the medium EBSS for 1 h. The cells were lysed and two hundred microliters of the cell lysates were loaded on the top of continuous sucrose gradients (0–30%). The samples were sedimented at 70,000*g* for 16 h at 4 °C with a preparative ultracentrifuge (CP80WX, Hitachi, Japan), and then all fractions were collected from the top of the gradient. Fractions were subjected to Western blotting analysis using the indicated antibodies.

### Gel filtration

HeLa cell lines (RAB37 overexpression, RAB37 knockdown and control HeLa cells) and two transiently transfected cells (FLAG-RAB37-Q89L, FLAG-RAB37-T43N) were cultured in DMEM medium with 10% FBS and starved in the medium EBSS for 1 h. The cells were lysed and then centrifuged at 50,000*g* for 30 min. The supernatants (cytosol fraction) were filtered through 0.22 μm filter. Then the fractions were separated by size-exclusion chromatography on a Superose 6 increase 10/300 GL column (Cat# 29-0915-96, GE Healthcare Life Sciences, Fairfield, Connecticut, USA), which has a very broad fractionation range (Mr 5,000–5,000,000), especially for large proteins and complexes, and eluted at a flow rate of 0.5 ml min^–1^ using the solution (50 mM sodium phosphate, 150 mM sodium chloride, pH 7.2). Fractions were subjected to Western blotting using relevant antibodies. The column was calibrated with gel filtration protein standards (Cat# 28-4038-42, GE Healthcare Life Sciences, Fairfield, Connecticut, USA) containing thyroglobulin (669 kDa), ferritin (440 kDa), aldolase (158 kDa), Conalbumin (75 kDa), Ovalbumin (43 kDa).

### Immunofluorescence analyses

COS-7 and HeLa cells were cultured on glass coverslips. At 48 h after transfection using FuGENE HD, the cells were starved in EBSS for 1 h. The tissues were cut using a freezing microtome (Leica, CM1850, Wetzlar, Germany). The cells and tissue sections were fixed with methanol for 20 min at −20 °C and permeabilised with 0.1% Triton X-100 in PBS for 30 min. After treating with 5% BSA for 30 min at room temperature, the samples were incubated with primary antibody overnight at 4 °C. After washing with PBS 5 times, the cells were subjected to indirect immunofluorescence secondary antibody. The nuclei were stained with DAPI. Images were captured with a confocal fluorescence microscope (Olympus, FV1000, Tokyo, Japan).

### Immune electron microscopy

HeLa cells were cultured in DMEM medium with 10% FBS. After transfected with pCMV-Tag2B or FLAG-RAB37, the cells were cultured in EBSS for 1 h, fixed with 4% paraformaldehyde and 0.25% glutaraldehyde for 4 h at 4 °C in 0.1 M sodium-phosphate buffer (pH 7.4). For transmission electron microscopy, cell samples were fixed with a 0.1 M sodium-phosphate buffer (pH 7.4) containing 2.5% glutaraldehyde overnight at 4 °C and fixed in 1% osmium tetroxide for 2 h at 4 °C. Following a stepwise ethanol and acetone dehydration and infiltration with Spurr’s epoxy resin, the samples were embedded and polymerized in Spurr’s epoxy resin at 60 °C for 48 h, and then sectioned at a thickness of 70 nm. The samples were blocked for 1 h with 1% BSA (0.05 M TBS, pH 7.4) at 25 °C, incubated with anti-FLAG antibody (0.1% BSA, 0.05 M TBS, pH 7.4) overnight at 4 °C, and washed (0.05 M TBS, pH 7.4) three times at 25 °C. Then the samples were incubated for 2 h at 25 °C with an anti-mouse IgG conjugated to 6 nm gold particles (Cat# 715-195-150, Jackson ImmunoResearch Laboratories, West Grove, PA, USA) (0.1% BSA, 0.05 M TBS, pH 8.2), washed (0.05 M TBS, pH 8.2) four times at 25 °C, and washed three times with miniQ H_2_O at 25 °C. The samples were double stained with 2% uranyl acetate and Sato’s lead citrate and analysed with JEM-1400 plus transmission electron microscope (JEOL, Akishima, Tokyo, Japan).

### Transfection of siRNA oligonucleotides

siRNA oligonucleotides were synthesised from Genepharma, ShangHai, China. siRNA sequences targeting ATG5 [42]: siRNA 1#, 5′-GGUUUGGACGAAUUCCAACUUGUUU-3′; siRNA 2#, 5′-GAUCACAAGCAACUCUGGAUGGGAU-3′ and siRNA 3#, 5′-GCCAUCAAUCGGAAACUCAUGGAAU-3′; negative control RNA, 5′-UUCUCCGAACGUGUCACGUTT-3′. The siRNAs were transfected into HeLa cells using Mission siRNA Transfection Reagent (Cat# S1452, Sigma-Aldrich, St Louis, MO, USA).

### GST pull-down assays

BL21 cells were transformed with GST, GST-ATG12, GST-ATG5-WT, GST-ATG5-C, GST-ATG5-E or His-RAB37 respectively. Cells were cultured at 37 °C with 220 rpm and induced with 0.1 mM IPTG at 16 °C for 24 h. Supernatants (GST, GST-ATG12, GST-ATG5-WT, GST-ATG5-C, GST-ATG5-E) were mixed with glutathione-agarose beads (Cat# P2020, Solarbio, Beijing, China) for 4 h at 4 °C. The beads were washed three times with NETN lysis buffer. His-RAB37 supernatants were incubated with 50 µM GTPγS or DMSO control for 4 h at 4 °C. Then the beads were incubated with the supernatants (His-RAB37 + 50 µM GTPγS or DMSO control) and rotated at 4 °C overnight respectively. Finally, the glutathione-agarose beads were washed three times with NETN buffer, denatured by boiling for 10 min, and then subjected to SDS/PAGE and Western blots with antibodies.

### Yeast two-hybrid assay

The ATG5 and RAB37 constructs were transformed into yeast strain Y2HGold using the quick and easy yeast transformation mix (Cat# 631851, Clontech Laboratories, Inc. Takara Bio Company, Kyoto, Japan). To test the autoactivation and toxicity, yeast transformed with 100 ng of vectors (BD-ATG5, BD-ATG5-C, BD-ATG5-E) were grown on SD/-Trp/X (SD/-Trp supplemented with 50 μg/mL X-α-Gal), or SD/-Trp/X/A (SD/-Trp supplemented with 50 μg/mL X-α-Gal and 200 ng/mL aureobasidin A) at 30 °C for 2–4 days. Meanwhile, yeasts transformed with 100 ng of vector (AD-RAB37) were grown on SD/-Leu/X (SD/-Leu supplemented with 50 μg/mL X-α-Gal), or SD/-Leu/X/A (SD/-Leu supplemented with 50 μg/mL X-α-Gal and 200 ng/mL aureobasidin A) at 30 °C for 2–4 days. The growth of white colonies on SD/-Trp/X or SD/-Leu/X media and no colony growth on SD/-Trp/X/A or SD/-Leu/X/A plates could indicate no autoactivation and toxicity. Then the constructs were cotransformed into yeasts and were selected on SD/-Leu/-Trp plates. Interaction between two proteins was monitored by growing on SD/-Leu/-Trp/-His plates at 30 °C.

### Co-localization analysis

Plasmids with different fluorescence were co-transfected into COS-7, or HeLa cells. Images were taken by the confocal fluorescence microscope (Olympus, FV1000, Tokyo, Japan). Co-localized dots were counted and data were prepared as Excel files. Data were expressed as means ± SD. T-test was used for the statistic analysis. * stands for *P* < 0.05; ** stands for *P* < 0.01.

### Statistics analysis

The data are presented as the means ± standard deviation. Statistical comparisons were made using Student’s *t*-test when comparing two groups. One-way analysis of variance (ANOVA) with Tukey’s post hoc test was performed for comparisons among more than two groups. These analyses were performed using the statistical package IBM spss statistics software (SPSS) version 22. For all analyses, a *P* value < 0.05 was considered to be statistically significant.

### Cell proliferation and scratch migration analysis

The cells were cultured in 12-well plates. The cell proliferation rate was determined using a cell counting chamber. Every 12 h, the cells from each well were collected and counted. For scratch migration analysis, the cells were cultured in 12-well plates, and a gap on the surface of the well was scratched with a 10-µl tip. After culturing for 12 h, the relative cell migration distance was used to compare the migration rates between cell lines. Eight tests for every cell line were performed.

### Soft agar assay for colony formation

A soft agar assay for colony formation was used to examine the cellular proliferation ability between lines. The cells were cultured between a 0.6% soft agar layer and a 0.35% soft agar layer; all agar layers contained 10% FBS and 100 units/mL penicillin and 100 µg/mL streptomycin. After 2 weeks, the clone size was measured. The total clone numbers and the clone area of each plate were calculated using the software Fuji (Japan).

### Tumour xenografts

Three cell lines (RAB37 overexpression, RAB37 knockdown and control HeLa cells) were suspended in normal saline and implanted into nude mice to generate tumours. Five mice per six were prepared for each cell line, and 2 × 10^6^ cells were implanted per mouse. After 4 weeks, the nude mice were sacrificed, and the tumours were collected for subsequent experiments.

## Electronic supplementary material

Supplementary material
