# Supplementary material for: RAB37 interacts directly with ATG5 and promotes autophagosome formation via regulating ATG5-12-16 complex assembly
Source: Cell Death Differ. 2017 Dec 11;25(5):918–34. doi: 10.1038/s41418-017-0023-1 (PMC5943352; doi:10.1038/s41418-017-0023-1)
Supplement: Supplementary file 1 — Supplementary material [file 41418_2017_23_MOESM1_ESM.docx]

**Supplementary information (3 Figures)**

**Figure 1**

**
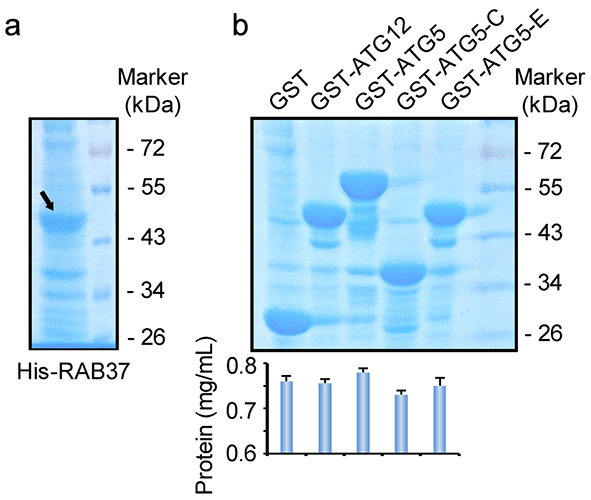
**

**Figure 1** Expression and quantification of recombinant proteins for GST pull-down assays. (**a**) Coomassie gel showed expression of His-RAB37 protein in supernatant of *E. coli* culture. (**b**) Coomassie gel showed expression of GST, GST-ATG12, GST-ATG5-WT, GST-ATG5-C and GST-ATG5-E in supernatant of *E. coli* culture. Supernatants were subjected to SDS/PAGE, and were detected by coomassie brilliant blue R250 staining. Right: The gel was calibrated with molecular weight of protein standards. Meanwhile, the concentration of all recombinant proteins (His-RAB37, GST, GST-ATG12, GST-ATG5-WT, GST-ATG5-C, GST-ATG5-E) were determined by the Pierce BCA protein assay kit (**b**).

**Figure 2**

**
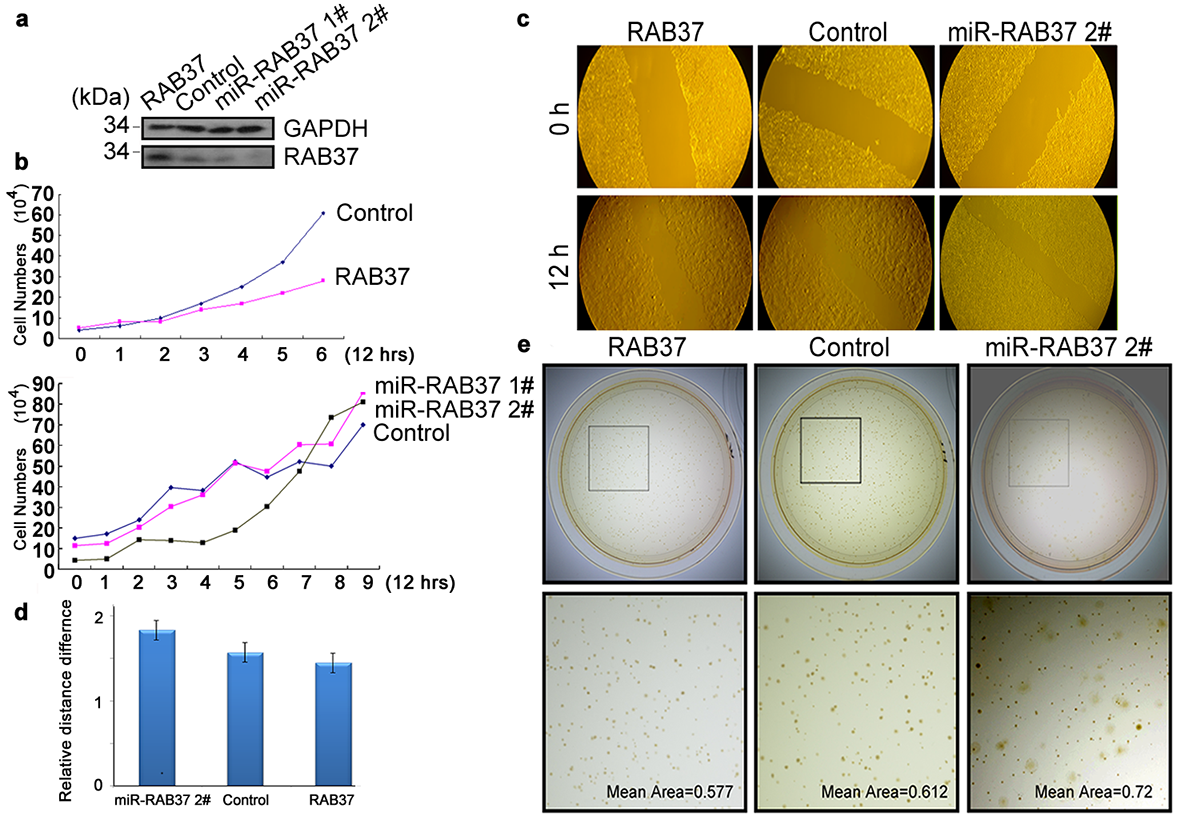
**

**Figure 2** RAB37 prevents cell proliferation and migration. (**a)** Determination of effective RAB37 knockdown vectors. Cell lysates derived from HeLa cells (control) and HeLa cells stably expressing CMV-RAB37, miR-RAB37 1# and miR-RAB37 2# were analysed using immunoblotting with an anti-RAB37 antibody. Knockdown in miR-RAB37 2# is more obvious than miR-RAB37 1#. GAPDH served as an endogenous control. The molecular marker is shown on the left (kDa). (**b)** RAB37 knockdown promotes and overexpression inhibits cell growth. Cell counting was used to examine the cellular proliferation rate in the RAB37 overexpression, knockdown and control (vector only) cells at the indicated times. The cell numbers were calculated every 12 hrs. (**c** and **d)** Scratch migration test. RAB37 knockdown promotes cell migration. The cells were cultured on 12-well plates for 12 hrs and then scratched with a 10-µl tip. After 12 hrs, the relative migration distance was examined. Statistical analyses from eight tests for each cell line were performed; the error bars indicate the standard deviation. (**e)** Soft agar assay for colony formation. The cells were cultured between two soft agar layers. Higher magnification views of the boxed areas are shown on the bottom. The mean relative areas per clone are shown in each panel.

**Figure 3**

**
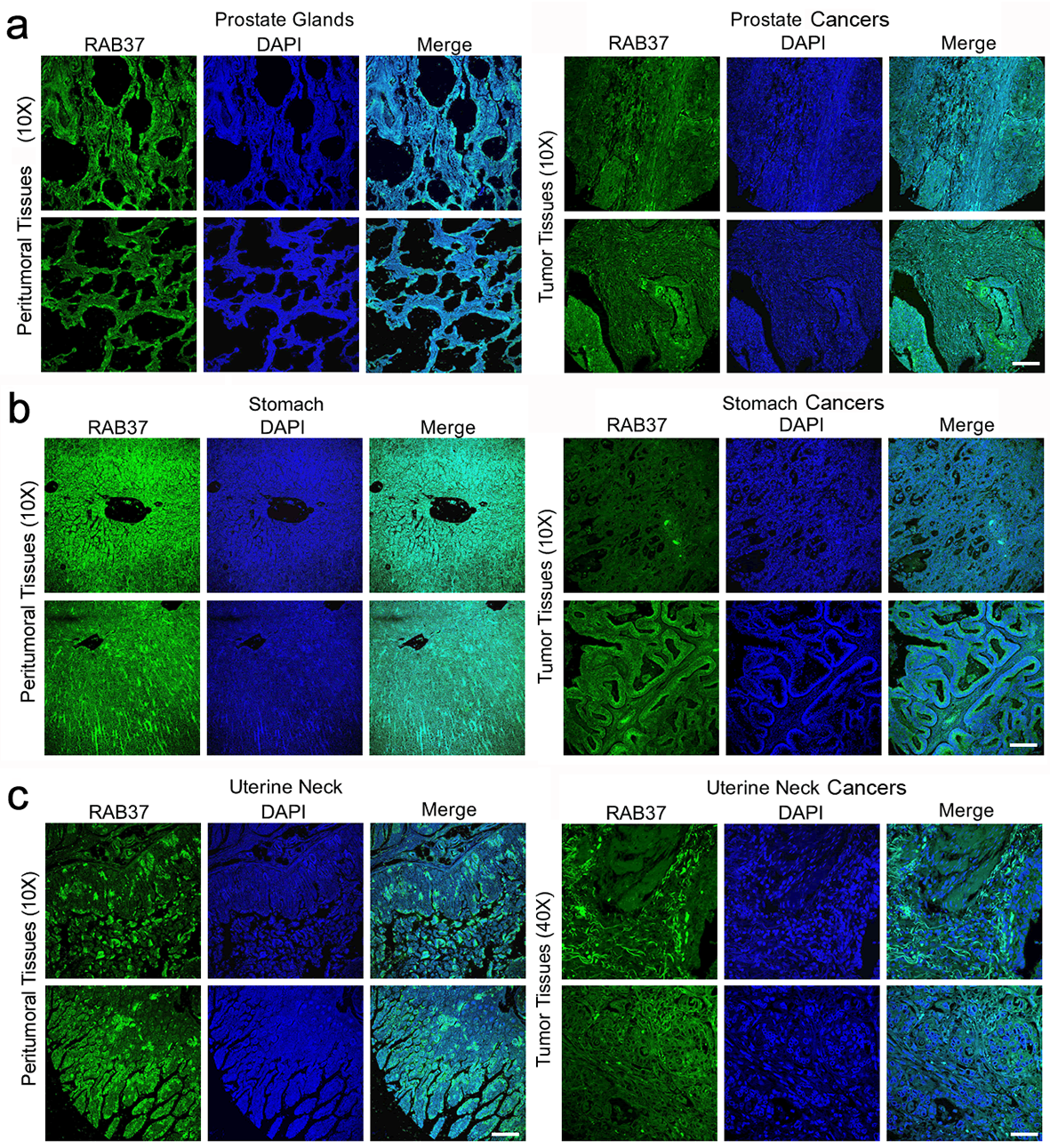
**

**Figure 3** Down-regulation of RAB37 expression in human tumors. The tumor sections were immunostained with an anti-RAB37 antibody (FITC). The nuclei were counterstained with DAPI (blue). The images were captured using confocal microscopy. (**a)** Prostate gland peritumoral tissues, prostate adenocarcinoma (T3N2M1c) and prostate transitional cell carcinoma (T2N0M0). Scale bar, 200 nm. (**b)** Stomach peritumoral tissues, stomach adenocarcinoma (T3N0M0) and stomach adenocarcinoma (T3N1M0). Scale bar, 200 nm. (**c)** Uterine neck peritumoral tissues, cervical squamous cell carcinoma (T1b2N0M0) and cervical squamous cell carcinoma (T1bN0M0). Scale bar, 200 nm (10X) or scale bar, 50 nm (40X).
